# Experiences of families of public safety personnel: a systematic review protocol of qualitative evidence

**DOI:** 10.1186/s13643-021-01807-1

**Published:** 2021-09-27

**Authors:** Janette Leroux, Rachel Richmond, Sara Fitzpatrick, Hannah Kirkland, Deborah Norris, Alyson Mahar, Joy MacDermid, Rachel Dekel, Heidi Cramm

**Affiliations:** 1 School of Rehabilitation Therapy, Queen’s University, Kingston, Canada; 2 Department of Family Studies and Gerontology, Mount Saint Vincent University, Halifax, Canada; 3 Department of Community Health Sciences, University of Manitoba, Winnipeg, Canada; 4 School of Physical Therapy, Western University, London, Canada; 5 The Louis and Gabi Weisfeld School of Social Work, Bar-Ilan University, Ramat-Gan, Israel

**Keywords:** Family, Public safety, Relationships, Qualitative study

## Abstract

**Background:**

Public safety occupations are well-recognized to be dangerous and stressful. Despite recent attention on post-traumatic stress injuries among public safety personnel, there has been considerably less attention paid to the ongoing ways in which the risks and requirements associated with those occupations shape family life, and how families respond and adapt to those lifestyle dimensions. This systematic review aims to understand how day-to-day family life is affected and shaped when a family member works in a public safety sector, such as fire, police, paramedic, corrections, and emergency communications.

**Methods:**

Qualitative studies that examine the experiences of families or family members of public safety personnel will be included in this review, with no date or language restrictions. An initial search of Embase and CINAHL will be conducted, followed by an analysis of text words contained in the title and abstract, and of the index terms used to describe the articles. Databases to be searched for published studies include MEDLINE, Embase, Web of Sciences, CINAHL, PsycINFO, and Sociological Abstracts. Titles and abstracts will be screened by two independent reviewers. The full texts of selected studies will be assessed in detail, and findings and their illustrations will be extracted and aggregated. Any disagreements between the reviewers that arise at each stage will be resolved through discussion, or by a third reviewer. Further analysis of the synthesized findings will be informed by family systems theory.

**Discussion:**

The ways that occupational risks and requirements shape family life have been better investigated within other high-risk occupation groups, which has led to productive advancements in organizational policies and supports in the respective sectors. An understanding of the experiences which typify family life ongoing within PSP sectors is a critical gap in the development of meaningful family-informed occupational initiatives and supports.

**Systematic review registration:**

Submitted to PROSPERO for systematic review registration: CRD42020208126

**Supplementary Information:**

The online version contains supplementary material available at 10.1186/s13643-021-01807-1.

## Background

Public safety personnel (PSP) work in sectors like fire, police, paramedic, corrections, and emergency communications services [1]. Public safety careers have been described as a “high-risk lifestyle” where PSP work in “an environment wrought with extraordinary and persistent demands that are often cumulative in nature” [2]. Research suggests that PSP experience mental health issues at higher rates than the general population [3, 4]. The stress PSP face in their career is multi-dimensional and complex in nature [5] relating to the operational demands and occupational conditions of public safety work. Amidst recent attention on post-traumatic stress injuries arising from exposure to potentially traumatic events in the line of duty among PSP, there has been considerably less attention in understanding PSP mental health from a relational perspective, beyond the individual experience or clinical outcomes.

Within the limited PSP research that exists, the family emerges as a relevant sociological unit of study: for example, via work-family conflict [6], the spillover effect on the entire family [7, 8], and consequences on spousal relationships [9] and spousal well-being [10, 11]. This body of research also suggests that families play an important role in maintaining the mental health of the PSP [12]. However, while PSP are often provided with formal and informal supports to help with stressful experiences on the job, the families of PSP do not receive the same resources [13]. The research on the experiences of PSP family members in managing occupational risks and requirements is yet to be synthesized, particularly from the family members’ perspective. Using family members as the starting point for understanding how PSP occupations affect family life and how family functioning supports PSP in addressing their occupational challenges will yield rich insights into responses and adaptations associated with the unique dimensions of a PSP lifestyle. Such attention to the needs of families of PSP has the potential to simultaneously improve mental health and well-being PSP and their families.

Our study question is as follows: *what are the experiences of families of public safety personnel as they navigate the implications of occupational risks and requirements of public safety work?* Our study aims to identify the most common lifestyle dimensions characteristic of a career in public safety, the ongoing ways in which the risks and requirements associated with those occupations shape family life, and how families respond and adapt to those lifestyle dimensions. Application of family systems theory [14] and human ecology theory [15] will allow for a fulsome analysis of the complexity of family experiences.

## Methods

The proposed systematic review will be conducted in accordance with the Joanna Briggs Institute methodology for systematic reviews of qualitative evidence [16]. This protocol has been registered in PROSPERO (CRD42020208126) and is being reported in accordance with the reporting guidance provided in the Preferred Reporting Items for Systematic Reviews and Meta-Analyses Protocols (PRISMA-P) statement [17] (see checklist in Additional Table 1).

### Preliminary search

A preliminary search of PROSPERO, EPISTEMONIKOS, Campbell Collaboration, Joanna Briggs Institute Database of Systematic Reviews and Implementation Reports, and OpenGrey was conducted from July 2–6, 2020. The search found several reviews concerning mental health among different public safety sector populations; however, there were no qualitative systematic reviews focused on the experiences of the families of PSP. There was one scoping study identified, which focused on documenting the mental health outcomes of families of first responders. Our use of the term “public safety personnel” is validated by the Canadian Institute for Public Safety Research and Treatment [18], to be more broadly inclusive of personnel who ensure the safety and security of Canadians: PSP include, but are not limited to, border services officers, public safety communications officials, correctional workers, firefighters (career and volunteer), Indigenous emergency managers, operational intelligence personnel, paramedics, police (municipal, provincial, federal), and search and rescue personnel. Hence, no previous or current systematic reviews on the topic have been identified, and there is a lack of qualitative evidence specifically identifying the experiences of families in the context of public safety occupations. Although previous studies indicate an important role for families in the mental health and well-being of PSP and the impacts that occupational stress and trauma can have on families of PSP, very little is known about the families’ experiences themselves. This review will address these gaps by appraising and synthesizing all available evidence related to day-to-day family life as it is affected and shaped by a member of the family working as a PSP.

### Inclusion criteria—participants

This review will consider qualitative studies that examine the experiences of family members of PSP, with a broad definition of family, to include spouse or committed partner, children, sibling, or parent. Studies will also be included if they examine stress, coping, resource mobilization, or resiliency of the family unit or family system. There will be no age limits for family members.

Studies focused on the experiences of PSP as individuals will be excluded because of the limited relational insights that can be garnered from individual psychology, and particularly psychological phenomena with a clinical emphasis. While the PSP is a member of the family and may participate in research to provide their perception of the family’s experience in relation to the occupational risks and requirements of public safety work, the current study is seeking to elevate the often under-represented experiences of non-serving PSP family members. The exception to this is dual-serving families, where two members of a family are PSP. Studies which explore the perspectives on the family lives of these individuals will be included.

This review will not include studies that explore:

- Soley the perspective of the PSP;
- Court cases or family custody hearings;
- Military personnel, including prisoners of war, military police, or military families; or
- The experiences of families of patients or community members whom the PSP are serving.

The context of this review are all public safety sectors, including expansive definitions of occupational job types and roles within fire, police, paramedicine, communications, and corrections sectors. For example, our study will include not just police officers, but police analysts, detectives, inspectors, law enforcement specialists, etc.

### Types of studies

This review will consider studies that focus on qualitative data, including, but not limited to, designs such as phenomenology, grounded theory, ethnography, qualitative description, action research, and feminist research. Case studies, case reports, autoethnographies, or discourse analyses will not be included. No geographical or publication date limitations will be imposed. Studies published in English will be included in this review as this is the primary language of the reviewers. This review will consider qualitative studies within any disciplinary field.

### Search strategy

The search strategy aims to locate published studies by searching academic databases and hand searching reference lists for studies not identified through the original search.

An initial, limited search of Embase and CINAHL was undertaken using terms related to the phenomena of lifestyle, family life, and everyday stressors of occupational origin [“stress”, “spillover”, “family-work”, “work-family”, “lifestyle”, “strain”, “adaptation”, “resilience”, “demands”]. After extensive deliberation and iterations of these and other psych-social constructs relating to stress and family, we determined that the phenomenon was too elusive, and that, by introducing the above terms, we were limiting our scope by what we were able to conceptualize based on previous literature discovery and familiarity in the field. Accordingly, we re-focused our preliminary searching on the population and the context, with the experience that family and occupational grouping terms offering more discrete definitions with terms of shared meaning. We undertook a second round of searching to capture expansive terms and subject headings for the population (family) [“family system”, “family unit”, “guardian”, “family life”, “family relationships”, “family connection, “family support”, “living with”, “partner”, “spouse”, “wife/wives”, “husband”, “intimate partner”, “romantic partner” “intimate relationships”, “couple”, “children/child”, “kid/s”, “parent/s”, “sibling/s”], in combination with the context (public safety occupation) [“first responder”, “emergency service”, “emergency response”, “public safety personnel”, “police”, “firefighter”, “paramedic”, “communications”, “corrections”, etc]. This was followed by an analysis of the text words contained in the titles and abstracts and of the index terms used to describe the articles, which took place from July 13–31, 2020. A second search using the identified keywords and index terms will be undertaken with the aid of a librarian across all included databases, with unique search strategies tailored for each information source. An example search strategy for Embase is presented in Additional Table 2. The reference lists of all studies selected for critical appraisal will be screened for additional studies. Studies published in English will be included. No date or study geography limitations will be imposed on the search strategies.

### Information sources

The databases to be searched include Embase, MEDLINE, Web of Sciences, CINAHL, PsycINFO, and Sociological Abstracts.

### Study selection

Following the search, all citations will be collated and uploaded into EndNote X9 (Clarivate Analytics, PA, USA) and the duplicates removed. The titles and abstracts of articles identified in the search will be screened independently by two reviewers for assessment against the inclusion criteria for the review. Any disagreements between the reviewers that arise at each stage of the study selection process will be resolved through joint review and discussion, or by a third reviewer. Next, full citation details will be imported into the JBI System for the Unified Management, Assessment and Review of Information (JBI SUMARI; Joanna Briggs Institute, Adelaide, Australia). The full text of selected studies will be retrieved and assessed in detail against the inclusion criteria. Reasons for exclusion of full-text studies that do not meet the inclusion criteria will be recorded and reported in the systematic review. The results of the search will be reported in full in the final systematic review and presented in a Preferred Reporting Items for Systematic Reviews and Meta-analyses (PRISMA) flow diagram [19].

### Assessment of methodological quality

Studies meeting the inclusion criteria will be critically appraised for methodological quality by two independent reviewers, using the standard JBI Critical Appraisal Checklist for Qualitative Research [20]. The quality of studies included in the review will be considered in the analysis and will also be discussed in the findings and conclusion of the systematic review. Any studies with a methodological quality score of less than 6 out of 10 will not be included. Any disagreements that arise between the reviewers will be resolved through discussion, or with a third reviewer. Authors of papers will be contacted to request missing or additional data where clarification is required. The results of the critical appraisal will be reported in narrative form and in a table.

### Data extraction

Qualitative data will be extracted from papers included in the review using a standardized data extraction tool for qualitative evidence (JBI SUMARI). The extraction will be performed by two independent reviewers. The data extracted will include specific details about the population, context, culture, geographical location, study methods, and the phenomenon of interest relevant to the review question and specific objectives. Results will be cross-checked, and any differences discussed and clarified prior to entering data into JBI SUMARI. Findings and their illustrations will be extracted verbatim and assigned a level of validity or credibility, as recommended in the JBI Reviewer’s Manual [16]. “Unsupported” findings will be excluded from the review. Any disagreements relating to the credibility that arise between the reviewers will be resolved through discussion, or by a third reviewer. Authors of papers will be contacted to request missing or additional data where clarification is required.

### Data synthesis

Qualitative research findings will, wherever possible, be pooled using JBI SUMARI with the meta-aggregation approach [20]. This will involve the aggregation or synthesis of findings to generate a set of statements that represent aggregation by assembling and categorizing findings on the basis of similarity of meaning. These categories will then be used to produce a single comprehensive set of synthesized findings that can be used as a basis for evidence-based practice. Where textual pooling is not possible, the findings will be presented in narrative form.

### Data analysis

Following the synthesis of the data, further analysis of the synthesized findings will be informed by family systems theory [14] and its theoretical variant, the human ecological theory [15]. The integration of these theories will enhance and frame the data by bringing into view a focus on interdependence between and among systems, bidirectional transactions across systems, and equilibrium. The experiences of PSP families are complex and require well-established family theories to support them and align the results of this review.

### Assessing confidence in the findings

The final synthesized findings will be graded according to the ConQual approach for establishing confidence in the output of qualitative research synthesis and presented in a Summary of Findings [21]. The Summary of Findings includes the major elements of the review and details how the ConQual score is developed. Included in the Summary of Findings will be the title, population, phenomenon of interest, and context for the specific review. Each synthesized finding from the review will then be presented along with the type of research informing it, a score for dependability, credibility, and the overall ConQual score.

## Discussion

Our study will answer the following research question: *what are the experiences of families of public safety personnel as they navigate the implications of occupational risks and requirements of public safety work?* We will identify the most common lifestyle dimensions characteristic of a career in public safety, the ongoing ways in which the risks and requirements associated with those occupations shape family life, and how families respond and adapt to those lifestyle dimensions.

The ways that occupational risks and requirements shape family life have been better investigated within other high-risk occupation groups, which has led to productive advancements in organizational policies and supports in the respective sectors. For example, family military lifestyle stressors related to deployment have been characterized to include risk, parental separation (parental absence), and mobility [22–24]. At the same time, there is documentation that families of military personnel exhibit sophisticated coping strategies and demonstrate system resiliency amidst unique conditions of uncertainty and disruption [25]. The body of research on the shared experiences of military families has furthered consideration of family life within military decision-making and has translated into tangible improvements for the families and serving personnel [26].

An understanding of the experiences which typify family life ongoing within PSP sectors is a critical gap in the development of meaningful family-informed occupational initiatives and supports.

Potential limitations for the current review include the consideration of studies published only in the English language for inclusion and the restriction of literature searches to the selected databases. Results will be disseminated through publication in a peer-reviewed journal. Any amendments made to this protocol during the review will be outlined in PROSPERO and reported in the final manuscript.

## Supplementary Information

**Additional file 1: Table S1**: PRISMA-P 2015 Checklist. **Table S2**: Search strategy.

## Data Availability

Not applicable.

## Abbreviations

JBI: Joanna Briggs Institute
PSP: Public Safety Personnel
