# Supplementary material for: Experiences of families of public safety personnel: a systematic review protocol of qualitative evidence
Source: Syst Rev. 2021 Sep 27;10:258. doi: 10.1186/s13643-021-01807-1 (PMC8474829; doi:10.1186/s13643-021-01807-1)
Supplement: Supplementary file 1 — Additional file 1: Table S1: PRISMA-P 2015 Checklist. Table S2: Search strategy. [file 13643_2021_1807_MOESM1_ESM.docx]

**Additional Table 1: PRISMA-P 2015 Checklist.** This checklist has been adapted for use with systematic review protocol submissions to BioMed Central journals from Table 3 in Moher D et al [17].

| **Section/topic** | **#** | **Checklist item** | **Information reported** | | **Line number(s)** |
| --- | --- | --- | --- | --- | --- |
|  |  |  | **Yes** | **No** |  |
| **ADMINISTRATIVE INFORMATION** | | | | | |
| **Title** | | | | | |
| Identification | 1a | Identify the report as a protocol of a systematic review |  |  | 1-3 |
| Update | 1b | If the protocol is for an update of a previous systematic review, identify as such |  |  | N/A |
| **Registration** | 2 | If registered, provide the name of the registry (e.g., PROSPERO) and registration number in the Abstract |  |  | 28 |
| **Authors** | | | | | |
| Contact | 3a | Provide name, institutional affiliation, and e-mail address of all protocol authors; provide physical mailing address of corresponding author |  |  | Title Page – 5-22 |
| Contributions | 3b | Describe contributions of protocol authors and identify the guarantor of the review |  |  | 247-253 |
| **Amendments** | 4 | If the protocol represents an amendment of a previously completed or published protocol, identify as such and list changes; otherwise, state plan for documenting important protocol amendments |  |  | 24-25 |
| **Support** | | | | | |
| Sources | 5a | Indicate sources of financial or other support for the review |  |  | 238-246 |
| Sponsor | 5b | Provide name for the review funder and/or sponsor |  |  | 239-240 |
| Role of sponsor/funder | 5c | Describe roles of funder(s), sponsor(s), and/or institution(s), if any, in developing the protocol |  |  | 246 |
| **INTRODUCTION** | | | | | |
| **Rationale** | 6 | Describe the rationale for the review in the context of what is already known |  |  | 33-55 |
| **Objectives** | 7 | Provide an explicit statement of the question(s) the review will address with reference to participants, interventions, comparators, and outcomes (PICO) |  |  | 56 |
| **METHODS** | | | | | |
| **Eligibility criteria** | 8 | Specify the study characteristics (e.g., PICO, study design, setting, time frame) and report characteristics (e.g., years considered, language, publication status) to be used as criteria for eligibility for the review |  |  | 90-117 |
| **Information sources** | 9 | Describe all intended information sources (e.g., electronic databases, contact with study authors, trial registers, or other grey literature sources) with planned dates of coverage |  |  | 146-149 |
| **Search strategy** | 10 | Present draft of search strategy to be used for at least one electronic database, including planned limits, such that it could be repeated |  |  | Additional Table 2 |
| ***STUDY RECORDS*** | | | | | |
| Data management | 11a | Describe the mechanism(s) that will be used to manage records and data throughout the review |  |  | 173-175 |
| Selection process | 11b | State the process that will be used for selecting studies (e.g., two independent reviewers) through each phase of the review (i.e., screening, eligibility, and inclusion in meta-analysis) |  |  | 153 |
| Data collection process | 11c | Describe planned method of extracting data from reports (e.g., piloting forms, done independently, in duplicate), any processes for obtaining and confirming data from investigators |  |  | 173-182 |
| **Data items** | 12 | List and define all variables for which data will be sought (e.g., PICO items, funding sources), any pre-planned data assumptions and simplifications |  |  | 94-96, 103-106, 128 |
| **Outcomes and prioritization** | 13 | List and define all outcomes for which data will be sought, including prioritization of main and additional outcomes, with rationale |  |  | 90-93 |
| **Risk of bias in individual studies** | 14 | Describe anticipated methods for assessing risk of bias of individual studies, including whether this will be done at the outcome or study level, or both; state how this information will be used in data synthesis |  |  | 189, 202 |
| ***DATA*** | | | | | |
| **Synthesis** | 15a | Describe criteria under which study data will be quantitatively synthesized |  |  | 184 |
|  | 15b | If data are appropriate for quantitative synthesis, describe planned summary measures, methods of handling data, and methods of combining data from studies, including any planned exploration of consistency (e.g., *I* ^2^, Kendall’s tau) |  |  | N/A |
|  | 15c | Describe any proposed additional analyses (e.g., sensitivity or subgroup analyses, meta-regression) |  |  | 191 |
|  | 15d | If quantitative synthesis is not appropriate, describe the type of summary planned |  |  | 184 |
| **Meta-bias(es)** | 16 | Specify any planned assessment of meta-bias(es) (e.g., publication bias across studies, selective reporting within studies) |  |  | 198 |
| **Confidence in cumulative evidence** | 17 | Describe how the strength of the body of evidence will be assessed (e.g., GRADE) |  |  | 201 |

**Additional Table 2: Search strategy**

Specific search strategy used in Embase (Embase Classic + Embase) is shown below, from the dates 1947 to 2020 August 12. These search strings will be modified as appropriate with the aid of a librarian for the other databases mentioned in this protocol.

| **Search** | **Query** | **Records retrieved** |
| --- | --- | --- |
| 1 | public safety personnel.mp. | 45 |
| 2 | emergency personnel.mp. | 458 |
| 3 | emergency respon*.mp. | 4,470 |
| 4 | blue light*.mp. | 11,451 |
| 5 | detective*.mp. | 2,130 |
| 6 | constable*.mp. | 126 |
| 7 | deputy chie*.mp. | 58 |
| 8 | superintendent*.mp. | 658 |
| 9 | sergeant*.mp. | 277 |
| 10 | uniformed officer*.mp. | 4 |
| 11 | platoon*.mp. | 208 |
| 12 | auxiliary.mp. | 32,067 |
| 13 | rcmp.mp. | 125 |
| 14 | red serge.mp. | 0 |
| 15 | mountie*.mp. | 2 |
| 16 | federal police.mp. | 103 |
| 17 | northwest mounted police.mp. | 0 |
| 18 | r c m p.mp. | 1 |
| 19 | royal canadian mounted police.mp. | 58 |
| 20 | police.mp. | 22,932 |
| 21 | tactical population*.mp. | 9 |
| 22 | fire inspector*.mp. | 4 |
| 23 | fire code*.mp. | 18 |
| 24 | (fire and rescue*).mp. [mp=title, abstract, heading word, drug trade name, original title, device manufacturer, drug manufacturer, device trade name, keyword, floating subheading word, candidate term word] | 1,281 |
| 25 | marine rescue*.mp. | 2 |
| 26 | trench rescue*.mp. | 1 |
| 27 | high angle rescue*.mp. | 0 |
| 28 | hazmat.mp. | 231 |
| 29 | hazardous material*.mp. | 1,625 |
| 30 | international association of fire fighters.mp. | 6 |
| 31 | coast guard*.mp. | 313 |
| 32 | ambulance*.mp. | 21,000 |
| 33 | base hospital*.mp. | 923 |
| 34 | paramed*.mp. | 34,408 |
| 35 | emergency medical responder*.mp. | 34 |
| 36 | emergency medical service*.mp. | 14,252 |
| 37 | emergency medical technician*.mp. | 1,489 |
| 38 | ornge.mp. | 10 |
| 39 | air ambulance*.mp. | 814 |
| 40 | paramedic*.mp. | 29,145 |
| 41 | volunteer emergency service*.mp. | 3 |
| 42 | community first responder*.mp. | 39 |
| 43 | call taker*.mp. | 84 |
| 44 | dispatcher*.mp. | 1,114 |
| 45 | communications officer*.mp. | 12 |
| 46 | security classification.mp. | 13 |
| 47 | detention cent*.mp. | 720 |
| 48 | correctional cent*.mp. | 138 |
| 49 | penetentiar*.mp. | 3 |
| 50 | remand*.mp. | 530 |
| 51 | inmate*.mp. | 6,719 |
| 52 | parole*.mp. | 1,498 |
| 53 | statutory release*.mp. | 1 |
| 54 | probation*.mp. | 2,387 |
| 55 | case manager*.mp. | 5,040 |
| 56 | caseworker*.mp. | 409 |
| 57 | incarcerat*.mp. | 15,877 |
| 58 | doing time.mp. | 32 |
| 59 | correctional service*.mp. | 174 |
| 60 | program officer*.mp. | 81 |
| 61 | forensic service worker*.mp. | 0 |
| 62 | forensic nurs*.mp. | 735 |
| 63 | prison staff.mp. | 286 |
| 64 | correction* officer*.mp. | 213 |
| 65 | rescue personnel/ | 7,759 |
| 66 | halfway house/ | 1,461 |
| 67 | police/ or emergency police dispatcher/ | 13,204 |
| 68 | fire fighter/ | 3,149 |
| 69 | prisoner/ | 17,814 |
| 70 | or/1-69 | 189,557 |
| 71 | family life/ or family coping/ or family functioning/ or family interaction/ | 16,312 |
| 72 | family stress/ | 2,321 |
| 73 | family/ or adopted child/ or adoption/ or adult child/ or childlessness/ or dysfunctional family/ or family decision making/ or family life/ or family relation/ or family separation/ or parenthood/ or single-parent family/ | 138,170 |
| 74 | spouse/ or domestic partner/ or husband/ or wife/ | 19,633 |
| 75 | significant other*.mp. | 5,331 |
| 76 | exp marriage/ | 73,187 |
| 77 | couple*.mp. | 437,379 |
| 78 | co habit*.mp. | 399 |
| 79 | cohabit*.mp. | 6,670 |
| 80 | or/71-79 | 661,445 |
| 81 | 70 and 80 | 4,792 |
| 82 | limit 81 to (article or article in press or "review") | 3,808 |

Note: .mp stands for multi-purpose, * indicates that all possible terminations of the term will be included in the search, rcmp refers to the Royal Canadian Mounted Police, Mountie is a colloquially used term for the Royal Canadian Mounted Police, Ornge is a Canadian not-for-profit corporation and registered charity that provides air ambulance and associated ground transportation services for the province of Ontario, under the direction of the province's Ministry of Health.
